# Determinants of Maternal Behavior of Mobile Phone Use during Pregnancy

**DOI:** 10.1155/2020/9465019

**Published:** 2020-10-23

**Authors:** Min Li, Xia Wu, Guoqiang Sun, Min Peng

**Affiliations:** Department of Obstetrics, Maternal and Child Health Hospital of Hubei Province, Wuhan, China

## Abstract

Excessive use of mobile phones might bring negative physical and psychological consequences to pregnant women. This study aims to explore the potential determinants of pregnant women's mobile phone use behavior to assist healthcare providers in the development of guideline programs. In order to explain the behavior, we developed a theoretical model based on the widely applied theory of planned behavior (TPB) by incorporating two additional constructs of personal habit and perceived risk. Structural equation modeling technique is employed to estimate the model based on questionnaire survey. Research results clearly show that behavior attitude and perceived behavior control play dominant roles in determining the intention and behavior. It is interesting to find that perceived risk and personal habit are less important in determining pregnant women's behavior of mobile phone use. Finally, suggestions are put forward to reduce the risk of mobile phone use during pregnancy.

## 1. Introduction

In the past few decades, the rapid development of mobile Internet has changed people's lifestyle. Internet technology has been successfully applied to many industries, including, but not limited to, agriculture [1], retailing [2], catering [3], and transportation [4, 5], leading to the so-called “Internet+”. It is unsurprising that mobile Internet has substantially improved the health care level [6, 7]. Nowadays, mobile phone use is ubiquitous. We can use mobile apps for purchasing drugs from online pharmacies [8], making online appointment with doctors [9], online diagnosis and monitoring [10], etc. In spite of these advantages, the negative financial, social, and health impacts of mobile phone use also deserve our attention, especially for teenagers, pregnant women, and elderly people [11]. For instance, exposure to radio frequency fields is increasingly common, and fetuses and children may be more vulnerable than adults [12].

Due to thermal effects and nonthermal effects, excessive use of mobile phones by pregnant women may potentially increase the delivery risk for both mothers and fetuses [13]. The existing literature has shown that excessive use of mobile phones may be related to physical and psychological problems, such as anxiety, depression, social isolation, and lack of emotional and social skills [14–16]. Nevertheless, the previous literature has mainly focused on potential negative consequences but not on the study of the underlying influence mechanism of the maternal behavior of mobile phone use. It is very important to be overcautious to reduce mobile phone use before all the negative consequences have been clarified. To this end, this study aims to investigate the underlying mechanism that explains the maternal behavior of mobile phone use to guide healthcare providers to design effective intervene programs.

In order to prevent potential radiation risks, we have observed that a large number of pregnant women in China purchase radiation-proof clothing from large online platforms such as Jingdong and Taobao. However, in our daily life, many pregnant women around us highly rely on mobile phones to purchase products from online platforms, work, relax, and so on. The maternal behavior of excessive mobile phone use during pregnancy is inconsistent with its possible consequences due to the complexity of the determinants underlying the behavior. The maternal behavior of mobile phone use during pregnancy may be related to the understanding of potential risks, the lifestyle and habits before pregnancy, and the views of other important people. To propose effective interventions to mitigate the potential risks, it is urgent to explore the psychological determinants of maternal behavior of mobile phone use.

The topic of this study falls into the field of health education and health behavior that focuses on individual and social determinants of all kinds of health behaviors [17]. In the past two decades, the stream of this research has increased rapidly with the aim to improve public health [18, 19]. The topics that received dramatic attention include tobacco use, sedentary lifestyle, unhealthy diet, risky sexual behavior, drug use, and family and gun violence, etc [17, 20, 21]. To explore the impact mechanism of health behaviors, a large number of theoretical models, such as the Theory of Planned Behavior (TPB) [22], the Transtheoretical Model (TTT) [23], social network, and Health Belief Model (HBM) [17] have been successfully developed for predicting, explaining, and intervening health behaviors. In this study, we extend the widely investigated TPB model to empirically explore the psychological factors underlying the mobile phone use behavior by pregnant women. We choose TPB because it has been widely applied in studying individual behaviors. According to TPB, individual behavior intention is determined by three constructs: behavior attitude (BA), subjective norm (SN), and perceived behavior control (PBC) [22]. In TPB, BA is determined by an individual's negative or positive evaluation of performing the behavior, SN is the perception that other important people approve or disapprove of the behavior, and PBC is defined as a person's views on his or her ability and resource constraints to do the behavior [22]. In this study, BA is mainly measured by the perceived benefits in reducing the usage of mobile phones during pregnancy, SN is mainly related to the impact of the suggestions of pregnant women's family member and colleagues, and PBC reflects the barriers of one kind of specific behavior. For a pregnant woman, such barriers mainly indicate the possible negative impacts brought by reducing mobile phone use. With the development of information technology, a lot of activities in our daily lives, such as shopping, working, and relaxation, strongly rely on the apps installed on mobile phones. In this sense, it is not easy to reduce the usage of mobile phones.

It is well known that the traditional TPB model fails to consider other important factors, such as social identity and habits, which might be of great importance to specific research issues. In addition to the constructs included in TPB, we also incorporate personal habit (PH) and perceived risk (PR) into our theoretical model. We hypothesized that these two constructs could impose influences on both the intention and behavior of maternal mobile phone use during pregnancy. Meanwhile, it is noteworthy that the TPB model assumes that behavior intention is the direct predictor of behavior [22]. However, the transformation of intention to behavior may be relevant to other factors, such as the action planning and copping planning, habits, and environmental conditions [24, 25]. For example, even if a pregnant woman has a strong intention to reduce the mobile phone use, she may not reduce the usage of mobile phones during pregnancy due to the deeply developed habits in shopping or watching video programs prior to pregnancy. In addition, we observed that many pregnant women have limited knowledge on the possible risks associated with mobile phone use during pregnancy.

The previous introduction shows that the determinants of mobile phone use behavior is very complex, which motivates us to extend the classical TPB model to empirically explore the impact mechanism of maternal behavior of mobile phone use during pregnancy. To the best of our knowledge, TPB has never been applied to the maternal behavior of mobile phone use during pregnancy in the existing literature. Moreover, many researchers agree that TPB should be extended because of its future parsimony [24, 25]; we added PR and PH into TPB to address our research problem. An online questionnaire survey was conducted to measure the latent variables included in our theoretical model. Based on the confirmatory factor analysis, we used structural equation modeling (SEM) technique to explain how different factors affect the mobile phone use behavior. The results obtained show that PBC plays a dominant role in directly affecting the behavior of reducing mobile phone use, while personal habits significantly affect the intention of using mobile phone rather than affecting the behavior directly. In addition, the impact of perceived risks on the behavior is negligible. Based on the results, some suggestions are proposed to control and change pregnant women's behavior to promote public health.

The paper is structured as follows.  Section 2 introduces the constructs to develop a theoretical model. In Section 3, we will conduct a descriptive statistic analysis about our sample obtained from the questionnaire. In Section 4, the data are analyzed. In Section 5, structural equation modeling technique is employed to investigate the relationship between variables. Recommendations are provided based on the results.  Section 6 gives a conclusion and points out the limitations.

## 2. Literature Review, Hypotheses, and Theoretical Model

### 2.1. Literature Review

Professional maternal health services are important in improving maternal, fetal, and newborn health outcomes. It is estimated that 90% of pregnancy-related maternal deaths can be prevented with timely prevention [26]. It is of great importance for applying theoretical models to explore the psychological factors in order to control health behaviors, e.g., healthy diet, moderate physical activities and exercise, and avoiding heavy duties. In recent years, a large body of literature has focused on the prediction and explanation of maternal behaviors. Whitaker et al. [27] used the TPB to examine pregnant women's perceptions and intentions toward weight gain, physical activity, and nutrition through internet-based survey. Chitsaz et al. [28] examined the predictors of healthy eating behaviors with the application of TPB. Reference [29] also used TPB to predict healthy eating intention and adherence to dietary recommendation during pregnancy in Australia with a sample of 455 pregnant women through web-based nutrition questionnaire. A systematic review on the attitudes, barriers, and enablers to physical activity in pregnant women refers to [30]. In addition, numerous studies have shown that Theory of Reasoned Action (TRA), TPB, and Breastfeeding Self-Efficacy (BSE) framework can effectively identify relationships between maternal psychosocial factors and breastfeeding initiation [31]. Johnson-Young [32] used the TPB model to predict women's intentions to breastfeed newborns for 3 months, 6 months, and 1 year. They found that there exist important interactions between body satisfaction and attitudes based on a survey of 156 pregnant women. From above, we notice that there is a research gap in understanding the factors determining the behavior of maternal mobile phone use. In Section 2.2, we will develop a theoretical model to explain maternal mobile phone use behavior, which is fundamental for taking effective actions to control the behavior.

### 2.2. Hypotheses and Theoretical Model

TPB was proposed by Ajzen to explain individual behaviors based on TRA [22]. TPB is an extension of TRA by adding PBC as a determinant of behavioral intention. TRA and TPB both assume that the best predictor of a behavior is behavioral intention, which is in turn jointly determined by BA, SN, and PBC. In practice, TRA and TPB have been successfully applied to predict and explain a wide range of behaviors and intentions, including smoking, drinking, health services utilization, exercise, sun protection, breastfeeding, substance use, and HIV/STD prevention behaviors [17].

As can be seen from the literature, although there exist some potential risks of mobile phone use [15, 16], the underlying factors in determining the mobile phone use intention or behavior are still unclear, particularly for the pregnant women group, which impedes us to take effective prevention actions. In the classic TPB model, behavior intention is determined by BA, SN, and PBC, whereas for the maternal mobile phone use problem, we suppose that PH and PR may influence both intention and behavior. In the following, we will introduce the development of theoretical models by using TPB model in explaining maternal mobile phone use behaviors during pregnancy.

#### 2.2.1. Behavior Attitude

BA is mainly reflected by individual beliefs about the outcomes of performing a behavior [22]. In this study, we assume that a pregnant woman who holds the belief that reducing mobile phone use is beneficial to both mothers and children will tend to reduce use mobile phone during pregnancy. Actually, the benefits of reducing the use of mobile phones may have multiple aspects, such as reducing tiredness and fatigue, improving sleep quality, and reducing pressure [16]. It should be noted that moderate mobile phone use, which depends upon the content elements displayed on the mobile terminals, may have positive impacts to pregnant women. For example, a healthy and well-designed movie or music can make a pregnant woman feel pleasant. However, nowadays, many people strongly rely on mobile phones. They spend a lot of time browsing the Web, watching movies or video clips, checking Wechat moments, etc [33]. In such a case, BA plays a fundamental role in determining the maternal behavior of using mobile phones. Consequently, we assume that BA is positively related to the intention of reducing mobile phone use, which is formulated by the following hypothesis:

  H1: BA is positively related to the maternal intention of reducing mobile phone use during pregnancy.

#### 2.2.2. Subjective Norm

SN is defined as the individual perception whether people who are important to us approve or disprove performing a behavior [22]. A person's intention can be influenced by the views of other individuals. We tend to adopt the decisions which are recommended by others who are important to us, including friends, family members, teachers, classmates, and colleagues. In particular, as customer needs become more and more demanding and diversified products are provided on e-commerce platforms, customer choice confusion becomes more and more common, so other people's suggestions will become very important. Similarly, the maternal behavior of mobile phone use may be similarly affected by others. As mentioned, the causes of pregnancy fatigue are complex and the overuse of mobile phones can lead to physiological and psychological consequences. If a close friend of a pregnant woman strongly advises her to reduce mobile phone use, she will be likely to adopt the suggestion. In the existing literature, it is generally assumed that SN is positively related to intention [33]. The influence of SN on the maternal intention of mobile phone use is represented as the following hypothesis:

  H2: SN is positively related to the maternal intention of reducing mobile phone use during pregnancy.

#### 2.2.3. Perceived Behavior Control

Compared with TRA, TPB incorporates PBC as a new construct to represents people's actual control ability over behaviors [22]. In the literature, PBC is usually measured by the perceived ease and difficulty of a behavior, which are related to facilitators, barriers, opportunities, external requisite sources (e.g., time, money, skills, and cooperation relationship), etc. In particular, even if a specific action is known to be beneficial, there may be many obstacles and limitations in its execution. Even if a pregnant woman knows that excessive use of mobile phones can be harmful, it may be very difficult for her to reduce the usage of mobile phones because she strongly relies on mobile phones in daily activities such as work, shopping, and leisure. TPB assumes that PBC and intention jointly determine a behavior [22]. Therefore, we hypothesize as follows:

  H3: PBC is positively related to the maternal intention and behavior of reducing mobile phone use during pregnancy.

#### 2.2.4. Personal Habit

The previous constructs are defined psychologically. In addition, we suppose that the maternal behavior of mobile phone use may be closely related to PH. If a woman before pregnancy has developed the habit of using mobile phone to watch videos, engage in work, and browse the web before pregnancy, it will be very difficult to change her behavior after pregnancy in a short time. Actually, individual experience and habits can be included in the construct of PBC to reflect the barriers of performing a specific behavior. However, there exist significant differences between PBC and PH. PBC is mainly represented by the objective barriers or facilitators, while habits have already developed prior to pregnancy. It is noted that we suppose that the construct PH affects both intention and behavior. This is because the willing of reducing mobile phone use may be weak if a pregnant woman has developed deep habits. In addition, the construct PH also affects the maternal behavior of mobile phone use because a deeply developed habit cannot be changed easily even if the maternal intention of reducing mobile phone use during pregnancy is strong. Subsequently, the behavior will not be performed in practice. In addition, Ajzen [22] pointed out that past behavior is best treated not as a measure of habit but as a reflection of all factors that determine the behavior of interest, and the close relevance between past and later behavior is an indication of behavior's stability. However, in contrast to prior pregnancy, the physiological and psychological statuses of pregnant women during pregnancy have changed greatly. Thus we use PH as a new construct in the theoretical model to analyze its impact on intention and behavior. Actually, a lot of existing literature also treats habit as a new construct independent of the TPB model [34, 35]. We hypothesize that strong intention and good habit can be translated into the desired behavior. As a result, we propose the following two hypotheses:

  H4: PH is negatively related to the maternal intention and behavior of mobile phone use during pregnancy.

#### 2.2.5. Perceived Risk

Until now, the harm of low-frequency radiation to human health through nonthermal effect is still debated. It means that the potential impact mechanism of risk components is uncertain [13]. In this study, we consider PR as a construct to measure the possible negative consequences brought by all kinds of risk factors in mobile phone use. Meanwhile, Thomee [16] demonstrated that extensive mobile phone use could potentially lead to stress, sleep disturbance, and symptoms of depression. Since pregnant women are relatively vulnerable, the mental symptoms caused by mobile phone overuse may be more apparent. Through a follow-up statistical analysis, Divan et al. [12] also showed that the overuse of mobile phones was associated with children's behavioral difficulties, such as emotional and hyperactivity problems. In fact, in our interview, a proportion of participants think that there exist potential harms but some others are highly uncertain. In our theoretical model, we consider both the potential risks to both fetuses, e.g., hyperactivity and fetal abnormalities and leucocythemia, and the risks to pregnant women. If the PR is higher, a pregnant woman's intention will be stronger. To be general, we also assume that PR will simultaneously affect intention and behavior. Consequently, we represent the following hypothesis:

  H5: PR is negatively related to the maternal intention of mobile phone use during pregnancy.

**Figure 1:**
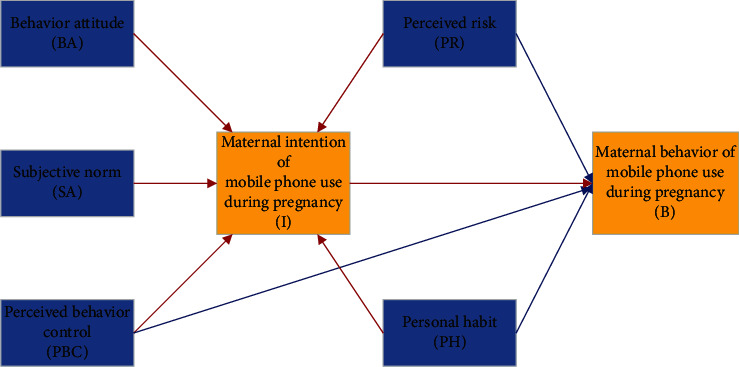
A theoretical model for studying the maternal behavior of mobile phone use during pregnancy.

Based on the previous hypotheses, a theoretical model for studying the maternal behavior of mobile phone use during pregnancy is developed, as shown in Figure 1. It shows that the intention of mobile phone use during pregnancy is determined not only by BA, SN, and PBC but also by PH and PR. The translation of intention into behavior is jointly determined by PBC, PH, and PR.

## 3. Procedure and Participants

Even though the literature on TPB is very rich, the items in the questionnaire should be designed specifically to the research problem. All the constructs are measured with multiple items, which are determined by face-to-face interviews with several pregnant women and obstetrical doctors. The questions asked in the interview mainly include the following:

1. What are the benefits of reducing mobile phone use during pregnancy?
2. Whose opinion do you think can positively affect a pregnant woman's behavior of mobile phone use during pregnancy?
3. What are the possible barriers of the maternal behaviors of mobile phone use during pregnancy?
4. What are the potential risks of mobile phone use during pregnancy to both fetuses and mothers?

The above four questions are associated with the constructs considered in our theoretical model. In the interview, the first three questions were mainly answered by pregnant women or the women who had pregnancy experience, while the last question was mainly answered by obstetric doctors.

In our questionnaire, questions are designed according to the measurement items, which are determined by the interview results, as represented in Appendix in Supplementary material. In the questionnaire survey, both pregnant women or the women who ever had pregnancy experience were included. Each participant is required to rate the extent to which they agree the statement by selecting a number from 1 to 7, in which 1 means “strongly disagree” and 7 means “strongly agree”. It means that our questionnaire adopts a 7-level Likert-type scale. The whole questionnaire process is administered through online survey. A total of 420 questionnaires were issued, while 387 answer sheets were used for this study, and the remaining answer sheets were eliminated due to data incompleteness and insufficient answer time.

Table 1 summarizes the basic information of the respondents. The respondents include both pregnant women and those who had pregnancy experience. Table 1 shows that most of them were aged between 25 and 35. The individuals in such an age group had witnessed the development procedure of mobile communication technology and their views were representative. Meanwhile, in terms of the education level, most of the respondents had obtained bachelor degree and the distribution of education level is reasonable. Generally speaking, the respondents who had been well educated will be more sensitive to the development of information technologies. We also collected the income and career data of our respondents. In terms of income and career, the sample is also representative because the sample is not limited to a specific group. In terms of the pregnancy stage distribution, we see that most of the respondents were in the third trimester or the childbirth had ended. The behavior data of these people were more reliable than those of pregnant women who were still in the first or second trimesters.

## 4. Data Analysis and Results

### 4.1. Measurements Model and Reliability Test

As introduced above, the candidate measurements were selected according to our theoretical model and the face-to-face interview. The construct BA was initially measured with four elements: reducing family conflict (Q10), relieving fatigue (Q11), improving sleeping quality (Q12), and keeping physically and mentally healthy (Q13). Family conflicts can be caused by overusing mobile phones because the opinions between different individuals are differentiated. For example, in China, a person who works very hard may dislike other family members use mobile phones extensively. They may think family members should have sufficient space and time for interacting with each other face-to-face. In this study, SN was mainly reflected by social opinion and the suggestions of family members, friends, and colleagues. The construct PBC was mainly measured by the barriers in working, social communication, relaxation, and so on. PH is measured by the intensity of past actions in shopping online, watching videos, listening music, and conducting job. PR mainly includes radiation, high-risk delivery outcomes to fetus, including fetal malformation, premature delivery, and possible pregnancy complications, such as pregnant hypertension, diabetes mellitus, and psychological problems. Finally, the maternal behavior of mobile phone use during pregnancy is measured by the actual frequency of mobile phone use during pregnancy. The intention is mainly evaluated by the future decisions made by pregnant women. For example, if a pregnant woman responds that she will try her best to avoid mobile phone use during pregnancy, we suppose that her intention is strong.

The measurements, which are shown in Table 2, were selected through confirmatory factor analysis in AMOS. For example, in the questionnaire, BA was initially measured with four elements: reducing family conflict (Q10), relieving fatigue (Q11), improving sleeping quality (Q12), and keeping physically and mentally healthy (Q13). However, we found that there exists strong collinearity between Q11 and Q12. Therefore, Q10, Q11, and Q12 were incorporated in our model. Similarly, other measurements were selected with the objective to measure the construct accurately. Finally, a total of 16 items were included in the measurement model for testing our theoretical model. The data reliability was analyzed by SPSS 19.0.  Table 3 shows that all the latent variables' Cronbach's *α* are above 0.7. It means that the data have a high degree of reliability. The KMO and Bartlett's test of sphericity have also been conducted to obtain the KMO test value of 0.899. Bartlett's test of sphericity is significant at the confidence level of 0.01, so the data are suitable for structural analysis.

## 5. Structural Model

**Figure 2:**
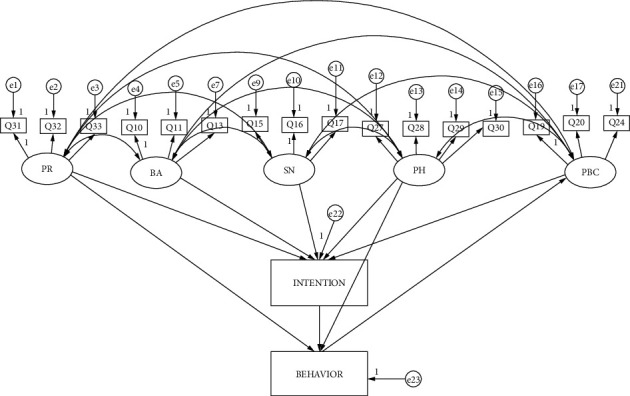
The structural model developed in this study.

Based on confirmatory factor analysis, we used structural equation modeling technique to test the hypotheses described in Section 2. In addition to the intention and behavior, the model includes five latent variables: BA, SN, PBC, PH, and PR. The structural model is represented in Figure 2.

The fitness, regression weights, and covariance in the structural model are computed with the AMOS software. To ensure that our model is capable of capturing the complex variable relationship, multiple metrics are selected to evaluate the fitness of the model: chi-square/degrees of freedom (*χ*^2^/d*f*), goodness-of-fit index (GFI), adjusted goodness-of-fit (AGFI), comparative fit index (CFI), and root-mean-square of approximation (RMSEA), etc. According to the standards introduced in the existing literature, the model fits the data well if *χ*^2^/d*f* < 3.0, CFI ≥ 0.9, GFI ≥ 0.9, NFI ≥ 0.9, AGFI ≥ 0.9, and RMSEA ∈ (0.05, 0.08) [36]. The fitness results of the model are shown in Table 4, from which we see that all the fitness metrics satisfy the requirement. It means that our model is capable of predicting maternal behavior of mobile phone use.

The method biases can influence the validity of the estimation results. To cope with the common method bias problem, both the design of study's procedures and statistical controls can be used. In this study, we performed Harman's single-factor test based on unrotated exploratory factor analysis. The results show a four-factor structure with the largest factor explaining 49% of the variables, less than the threshold of 50% [37]. In addition, we constructed a new model by adding a common method factor. The fitting performance is *χ*^2^/d*f*=3.352, CFI=0.952, GFI=0.907, RMSEA=0.078. Compared with the fitness results of the model depicted in Figure 2 (as shown in Table 4), the addition of method factor did not change the model fit significantly. Therefore, we conclude that the data do not suffer from serious common method bias.

**Figure 3:**
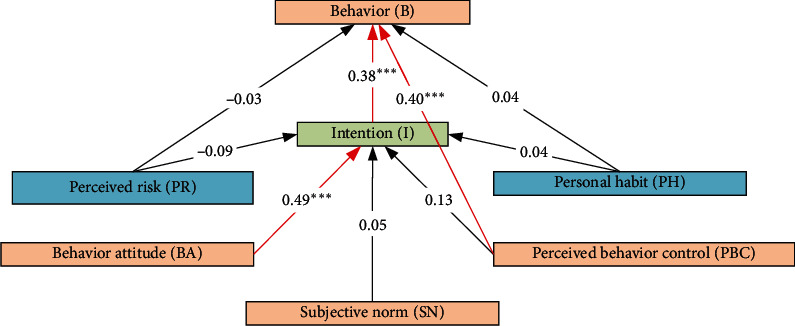
Model estimation using all data.

The estimation results of our model are shown in Table 5, in which UNSTD is the unstandardized estimates and STD is the standardized estimates. We see that most of the standardized parameter estimates between variables fall into the interval [0.35, 0.95]. The level of statistical significance was set at *p* < 0.05. From Table 5 and Figure 3, we can test the hypotheses included in our theoretical model.

We see that the C.R. values associated with hypothesis H1 are more than the reference value of 1.96. In addition, the relationships in H1 are statistically significant with *p* < 0.05. The standardized regression coefficient between BA and intention is 0.493. Thus, we can approve the hypothesis H1. It means that potential benefits can stimulate the intention of reducing mobile phone use. The benefits considered in the structural model include relieving family conflict (Q10), reducing fatigue (Q11), and regulating emotion (Q13). Nowadays, a lot of people take too much time in using mobile phones for different purposes, including social networking and relaxation. These behaviors may lead to physical tiredness and negative emotion. These consequences combining with the pregnancy reaction will certainly change the intention and behavior. The result also shows that reducing mobile phone use during pregnancy may be helpful for relieving the family conflicts. This can be explained by the intermediate influence of fatigue and emotion because physical tiredness and psychological status of a pregnant woman can readily affect the relationship in her family. Meanwhile, other family members' emotion can adversely propagate to the pregnant women in different forms, such as diet preparation and poor performance in working. From the perspective of intervention, on one hand, an obstetrician should encourage pregnant women to reduce the time of mobile phone use during pregnancy; on the other hand, an obstetrician could make pregnant women understand the possible negative influences and benefits brought by using mobile phones.

As mentioned, PBC refers to the ability, confidence, opportunity, and anticipated barriers in performing a specific action. For pregnant women, there are a lot of barriers for reducing mobile phone use, e.g., impeding work, hindering social communication, and reducing relaxation activities. In China, most pregnant women have short maternity vocation, and many women still need to work during pregnancy. Moreover, a lot of work needs to rely on mobile phone use. Table 5 and Figure 3 verify hypothesis H3 that PBC simultaneously affects both intention and behavior significantly. In the existing literature, some scholars have found that PBC mainly affects intention. In this study, it seems that PBC is more important for stimulating behaviors rather than strengthening intentions. Actually, individuals who have strong intention may not perform one specific action due to all kinds of constraints. This is also reasonable for pregnant women that intention does not directly translate into a behavior. The results on PBC provide us some important managerial implications. Firstly, the behavior of reducing mobile phone use during pregnancy may be closely related to career types and education levels. To relax the constraints imposed by work, employers can change pregnant women's job types temporarily or set long pregnancy vocations. Secondly, we should encourage pregnant women to communicate with others face-by-face rather than through online platforms and do outdoor exercises to eliminate the corresponding barriers.

Regarding SN, we found that its influences on both intention and behavior are relatively weak. It means that the impacts of the suggestions and views proposed by family members, friends, and colleagues are very limited due to individual differences. We also observed that PH is also not very important in translating intention to behavior. This is quite counter-intuitive according to the hypothesis H4, as we presume that pregnant women who watch online entertainment videos, communicate with friends mainly through online platforms, or purchase online frequently tend to last their habits. This is not true due to the significance of delivering a birth. Consequently, most of pregnant women will change their habits to delivery healthy babies. Finally, the results show that the influence of all kinds of risk factors, e.g., radiations, fetal diseases, and maternal diseases, are also not significant. The main reason for this result is because risky outcomes are highly uncertain. Actually, even if the antiradiation maternity suits sell well in e-commerce platforms, the impact of low-frequency radiations on both infants and mothers are still unclear. In addition, there is limited information on the association between mobile phone use and reproductive outcomes, e.g., spontaneous abortions, birth weight, and congenital malformation [16]. Since no established mechanism is known, it is impossible to exclude any health outcomes for consideration. In addition, maternal diseases are generally caused by other complex factors, such as immunity and heredity, age, weight, and lifestyles. However, we appeal that even though the risk of mobile phone use during pregnancy is highly uncertain, we still should expand the publicity of possible negative outcomes due to the high importance of pregnancy to both infants and mothers.

## 6. Conclusions

In this study, we explored the impact mechanism underlying the maternal intention and behavior of mobile phone use during pregnancy. We extended the TPB by adding two constructs PH and PR to consider the influence of habits and possible risks on the maternal behavior of mobile phone use [22, 24]. Although the possible negative consequences of mobile phone use have been investigated in the literature [14–16], from a psychological perspective, we pay attention to the underlying factors affecting the maternal behavior of mobile phone use. Confirmatory factor analysis is used to select the items to measure the constructs. Through questionnaire survey, we selected 387 samples for analysis. Based on the reliability analysis on our sample data, we constructed a structural equation model to test the hypotheses in our theoretical model.

The research results show that BA plays a dominant role in stimulating strong intention of reducing mobile phone use. In this regard, we should strengthen the publicity of the benefits brought by reducing the maternal behavior of mobile phone use, e.g., promoting family harmony, reducing tiredness, and stabilizing emotion. In addition, we also found that PBC simultaneously affects intention and behavior. This means that eliminating the possible barriers and searching for alternatives are important for reducing the mobile phone use behavior. We also obtained the result that the impact of PH is very weak. This may be attributed to the great significance of pregnancy to both infants and mothers. The lifestyles before pregnancy and after pregnancy can be substantially different. Finally, the results show that PR is relatively less important in leading to the maternal behavior of mobile phone use during pregnancy. This is due to that its negative consequences are highly uncertain or unknown to the public. The results obtained in this study can guide healthcare providers to reduce mobile use during pregnancy through training, encourage face-to-face communication, increase out-door exercises, read books, etc [38].

It should be mentioned that our research has some limitations that deserve further research in the future. Firstly, our sample is limited from a large hospital, which may introduce the risk of common method variance. The results obtained in this study should be validated with samples from different sources. Secondly, we did not compare our model with other existing theoretical models due to the shortage of literature. In addition, some other important factors, such as social identify, geographical, and environmental conditions, have been ignored in this study. A research direction deserving further exploration is to integrate different models to develop a comprehensive model, similar to the integrated model introduced in [17], to explain the maternal behavior of mobile phone use during pregnancy with consideration of many risk factors. Finally, our model is static by nature, but we gauge that pregnant women's intention and behavior evolve over time. The behavioral differences between different pregnancy stages and the underlying mechanism therein need to be investigated with other dynamic models, such as the Transtheoretical Model (TTT) [23].

## Figures and Tables

**Table 1:** Basic information of the respondents.

Attributes	Items	Frequency	Percent
Age	18–25	25	6.5
	25–30	169	43.7
	30–35	140	36.1
	>35	53	13.7

Education	College degree or below	111	28.7
	Bachelor	205	53.0
	Master	58	15.0
	Ph.D	13	3.3

Monthly income (yuan)	<8000	251	64.8
	8000–10000	75	19.4
	10000–15000	44	11.4
	15000–20000	7	1.8
	>20000	10	2.6

Career	Public servant	13	3.4
	Teacher	54	14.0
	Doctor	29	7.5
	Farmer	6	1.6
	Worker	20	5.2
	Self-employed worker	20	5.2
	Employment waiter	29	7.5
	Others	216	55.6

Pregnancy stage	Early pregnancy	12	3.1
	Pregnant metaphase	17	4.4
	Late pregnancy	168	43.2
	During pregnancy	190	49.1

Number of pregnancies	1	227	58.7
	2	98	25.3
	3	40	10.3
	>3	22	5.7

**Table 2:** Measurement items included in the structural model.

Constructs	Measurement items
Behavior attitude (BA)	Relieving family conflict (Q10)
	Reducing fatigue (Q11)
	Regulating emotion (Q13)

Subjective norm (SN)	Advices of family members (Q15)
	Advices of friends (Q16)
	Advices of colleagues (Q17)

Perceived behavior control (PBC)	Negative impact on social communication (Q19)
	Reducing social communication during pregnancy (Q20)
	Substitutions in relaxations (Q24)

Perceived risk (PR)	Radiations risks to fetus (Q31)
	Fetal disease risks, such as fetal abnormalities and leucocythemia (Q32)
	Causing maternal disease risks, such as hypertension and diabetes (Q33)

Personal habit (PH)	Shopping with mobile phone (Q27)
	Watching videos and listening music (Q28)
	Social communications on social platforms (Q29)
	Conducting jobs with mobile phones (Q30)

Intention (I)	Future intention on mobile phone use during pregnancy(Q9)
Behavior (B)	Frequency of using mobile phone (Q8)

**Table 3:** Results of reliability test.

Constructs	Items	Mean	S.D.	Cronbach's *α*
Behavior attitude (BA)	Q10	4.39	1.963	
	Q11	5.18	1.78	0.793
	Q13	4.34	1.85	

Subjective norm (SN)	Q15	5.11	1.683	
	Q16	4.79	1.75	0.936
	Q17	4.69	1.797	
	Q19	4.24	1.863	

Perceived behavior control (PBC)	Q20	3.57	1.951	0.75
	Q24	4.19	1.814	

Personal habit (PH)	Q27	3.36	2.002	
	Q28	3.31	1.969	0.892
	Q29	2.87	1.901	
	Q30	4.03	1.940	

Perceived risk (PR)	Q31	4.03	1.940	
	Q32	3.91	1.932	0.750
	Q33	3.44	1.931	

**Table 4:** The fitness results on the structural model.

Index class	Fitness metrics	Value	Standard
Absolute fitting index	*χ* ^2^	351.439	—
	*df*	118	—
	*χ* ^2^/*df*	2.978	<3
	RMSEA	0.072	<0.08
	GFI	0.909	>0.9

Relative fitting index	NFI	0.933	>0.9
	CFI	0.954	>0.9
	IFI	0.955	>0.9
	RFI	0.913	>0.9

Simple fitting index	PNFI	0.72	>0.5
	PGFI	0.627	>0.5

**Table 5:** The estimation results on the regression weights.

Effect	Cause	UNSTD	S.E.	C.R.	*p* value	STD
I	SN	0.062	0.074	0.834	0.404	0.054
I	PH	0.043	0.11	0.394	0.693	0.036
I	PBC	0.204	0.259	0.787	0.431	0.127
I	BA	0.623	0.161	3.876	*∗∗∗*	0.493
I	PR	0.099	0.079	1.259	0.208	0.09
Q31	PR	1				0.919
Q32	PR	1.002	0.035	28.871	*∗∗∗*	0.925
Q33	PR	0.856	0.041	20.911	*∗∗∗*	0.79
Q10	BA	1				0.795
Q15	SN	0.831	0.031	26.82	*∗∗∗*	0.84
Q17	SN	0.983	0.026	37.212	*∗∗∗*	0.931
Q27	PH	1				0.831
Q28	PH	1.004	0.051	19.642	*∗∗∗*	0.849
Q29	PH	0.95	0.05	19.09	*∗∗∗*	0.832
Q30	PH	0.898	0.052	17.278	*∗∗∗*	0.775
Q19	PBC	1				0.662
Q20	PBC	1.024	0.092	11.123	*∗∗∗*	0.648
B	I	0.341	0.05	6.82	*∗∗∗*	0.376
B	PR	−0.032	0.066	−0.481	0.631	−0.032
B	PBC	0.572	0.142	4.014	*∗∗∗*	0.395
Q13	BT	0.973	0.058	16.792	*∗∗∗*	0.821
Q16	SN	1				0.97
Q11	BA	0.714	0.058	12.323	*∗∗∗*	0.626
Q24	PBC	1.188	0.089	13.297	*∗∗∗*	0.808
B	PH	0.046	0.077	0.594	0.552	0.043

## Data Availability

The data will be available from the corresponding author through e-mail upon request.

## Supplementary Materials

Supplementary Materials Supplementary materials include the appendix questionnaire on pregnant women's mobile phone use behavior.
