# Supplementary material for: Determinants of Maternal Behavior of Mobile Phone Use during Pregnancy
Source: J Healthc Eng. 2020 Oct 23;2020:9465019. doi: 10.1155/2020/9465019 (PMC7603607; doi:10.1155/2020/9465019)
Supplement: Supplementary Materials — include the appendix questionnaire on pregnant women's mobile phone use behavior. [file 9465019.f1.pdf]

Appendix: Questionnaire on pregnant women's mobile phone use behavior

Hello! We are the obstetricians from Hubei Maternal and Child Health Hospital. We are now conducting a research on the psychological factors affecting the maternal behavior of mobile phone use during pregnancy. The following questionnaire will be completed voluntarily. The original data is used only for academic purpose and will be confidential to the public. If you agree to help us in this research, it will be highly grateful for you to continue to answer the following questions.

Part 1: Basic information

Please take a few minutes to let us know you. (Circle only ONE answer for the following questions.)

1. What is your age?

A. Below 18 B. 19-25 C. 26-30 D. 31-35 E. Over 35

2. What is your academic qualification?

A. Under college degree B. Bachelor C. Master D. Ph.D

3. What is your monthly salary (yuan)?

A. Under 3000 B. 3000-8000 C. 8000-10000 D. 10000-15000 E. Over 15000

4. What is your career?

A. Public servant B. Teacher C. Doctor D. Farmer

E. Worker F. Self-employed worker G. Employment waiter H.others

5. Which city are you living? _______

6. How many times of pregnancy experience do you have?

A. 1 B.2 C.3. D.>3

7.What is your pregnancy stage?

A. Early pregnancy B. Pregnant metaphase C.Late pregnancy D. After pregnancy

Part 2: Survey on the factors affecting behavior

Please take a few minutes to let us know your thoughts. In the following questions, you are required to select one number to indicate the extent of agreement, in which 1 denotes strongly disagree and 7 denotes strongly agree. The questions designed are shown in Table 6.

Table 6: Questions designed for understanding the maternal behavior of mobile phone use during pregnancy

Questions	Selectable Values						
**Q8.** I rarely use mobile phones during pregnancy.	1	2	3	4	5	6	7
**Q9.** I plan to reduce mobile phone use during pregnancy in future.	1	2	3	4	5	6	7
**Q10.** I think reducing mobile phone use during pregnancy can relieve family conflicts.	1	2	3	4	5	6	7
**Q11.** I think reducing mobile phone use during pregnancy can mitigating fatigue.	1	2	3	4	5	6	7
**Q12.** I think reducing mobile phone use during pregnancy can help improve sleeping.	1	2	3	4	5	6	7
**Q13.** I think reducing mobile phone use during pregnancy can make me feel happy.	1	2	3	4	5	6	7
**Q14.** I think the idea that pregnant women should reduce mobile phone use during pregnancy has become a social consensus.	1	2	3	4	5	6	7
**Q15.** My family members encourage me to reduce mobile phone use.	1	2	3	4	5	6	7
**Q16.** My friends encourage me to reduce mobile phone use.	1	2	3	4	5	6	7
**Q17.** My colleagues encourage me to reduce mobile phone use.	1	2	3	4	5	6	7
**Q18.** I think that reducing mobile phone use will not bring negative effects on my job.	1	2	3	4	5	6	7
**Q19.** I think reducing mobile phone use will not bring negative effects on my social communications.	1	2	3	4	5	6	7
**Q20.** I think pregnant women should reduce social communications during pregnancy.	1	2	3	4	5	6	7
**Q21.** I think the other family members of a pregnant woman also should reduce mobile phone use.	1	2	3	4	5	6	7
**Q22.** I think a pregnant woman can understand the social dynamics through reading newspapers and watching TV instead of using mobile phones.	1	2	3	4	5	6	7
**Q23.** I think reducing mobile phone use will not impede me to understand social changes.	1	2	3	4	5	6	7
**Q24.** I think reducing mobile phone use will not affect my relaxations.	1	2	3	4	5	6	7
**Q25.** I can increase outdoor exercises to avoid emptiness and boredom during pregnancy.	1	2	3	4	5	6	7
**Q26.**I can relax through reading books, listening music, and watching TV rather than using mobile phones.	1	2	3	4	5	6	7
**Q27.** I rarely use a mobile phone to shop prior to pregnancy.	1	2	3	4	5	6	7
**Q28.** I rarely watch videos and listen music by mobile phones.	1	2	3	4	5	6	7
**Q29.** I rarely communicate through mobile social network platforms, such as Wechat and QQ.	1	2	3	4	5	6	7
**Q30.** I rarely use mobile phone to work.	1	2	3	4	5	6	7
**Q31.** I think mobile phone use during pregnancy will produce negative radiations to fetuses.	1	2	3	4	5	6	7
**Q32.** I think that the overuse of mobile phones can bring negative outcomes to fetuses, e.g., fetal anomaly and premature fetus.	1	2	3	4	5	6	7
**Q33.** I think that overuse of mobile phones can bring negative outcomes to mothers, e.g., diabetes and hypertension.	1	2	3	4	5	6	7
